# Acute abdomen in the centanary patient, mesh migration into the sigmoid colon after laparoscopic inguinal hernia repair (TAPP): A case report and review of literature

**DOI:** 10.1016/j.ijscr.2019.11.050

**Published:** 2019-11-30

**Authors:** Roosevelt Fajardo, Francisco Diaz, Luis F. Cabrera, Mauricio Pedraza

**Affiliations:** a Department of General Surgery, Fundación Santa Fe de Bogotá, Bogota, Colombia; b Departmen of General Surgery, Universidad El Bosque, Bogota, Colombia

**Keywords:** Laparoscopic transabdominal preperitoneal (TAPP), Mesh, Erosion, Migration, Foreign body and acute abdomen

## Abstract

**Background:**

The complications induced by mesh, such as foreign body reaction, deep-seated infection, mesh migration and perforation into viscera, have been reported sporadically. Colon erosion and penetration by laparoscopic transabdominal preperitoneal (TAPP) inguinal hernia repair mesh can possibly cause perforation of the colon with acute abdomen.

**Case presentation:**

A 100-year-old male, who underwent 4 years ago TAPP repair of left inguinal, presented to the emergency department with acute abdomen due to chronic mesh penetration into the sigmoid colon, the migrating mesh generated a free wall perforation with generalized fecal peritonitis.

**Discussion:**

Tailoring the mesh, appropriate suture placement and adherence to principles of antisepsis during hernia repair surgery are crucial in avoiding longterm mesh-related complications.

**Conclusion:**

TAPP is a safe procedure for treat groin hernias, unless, mesh complications like foreign body reaction, deep-seated infection, mesh migration and perforation.

## Highlights

• Complications induced by mesh, such as foreign body reaction, deep-seated infection, mesh migration and perforation into viscera, have been reported sporadically.
• Colon erosion and penetration by laparoscopic transabdominal preperitoneal (TAPP) inguinal hernia repair mesh can possibly cause perforation of the colon.
• Migration of the mesh should suspect in patient with previous TAPP procedure.

## 1 Introduction

The complications induced by mesh, such as foreign body reaction, deep-seated infection, mesh migration and perforation into viscera, have been reported sporadically. The tension-free method with mesh as a muscle reinforcement technique is regarded as an important part of inguinal hernia repair since it reduces the hernia recurrence rate. With the introduction of laparoscopic inguinal hernioplasty the superficial infection rate has decreased dramatically (less than 2 %) [[1], [2], [3], [4]].

Chronic pain and surgical site infection associated with prosthetic mesh are well-known complications, which mainly occur in the early postoperative period. However, serious complications, such as mesh migration, erosion and perforation of adjacent organs, are rarely reported and may present symptoms at different time intervals after inguinal hernia repair [5,6].

Colon erosion and penetration by laparoscopic transabdominal preperitoneal (TAPP) inguinal hernia repair mesh can possibly cause perforation of the colon with acute abdomen. We report the first case of chronic mesh penetration into the sigmoid colon, where the migrating mesh generated a free wall perforation wiht generalized fecal peritonitis in a centenary patient [[7], [8], [9]]. This work has been reported in line with the SCARE criteria [27].

## 2 Case report

**Fig. 1:**
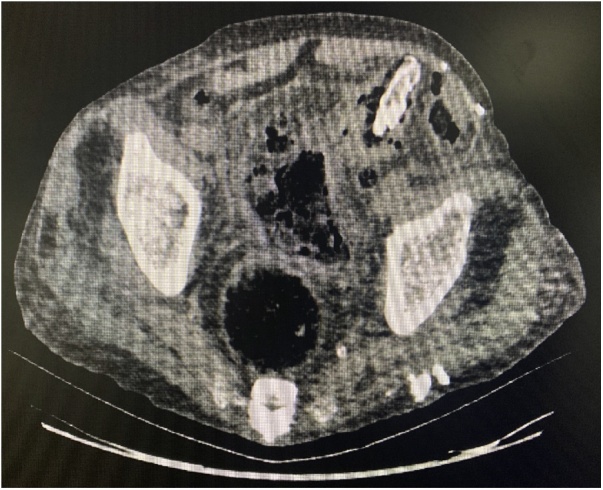
Abdominal CT scan with neumoperitoneum with a strange body into de left colon lumen.

**Fig. 2:**
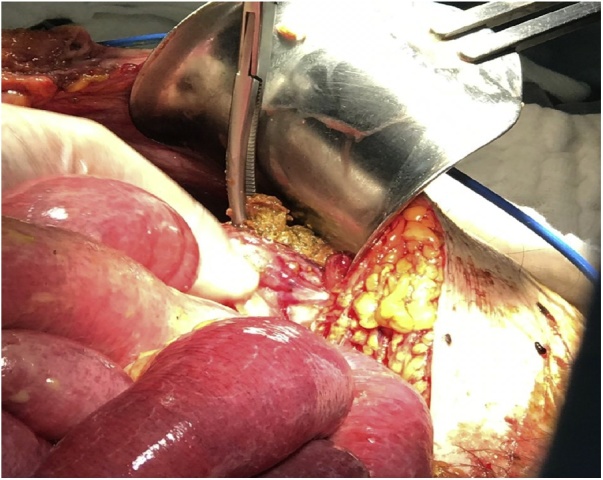
Left-sided hernia repair mesh penetrating the sigmoid colon from the preperitoneal space.

**Fig. 3:**
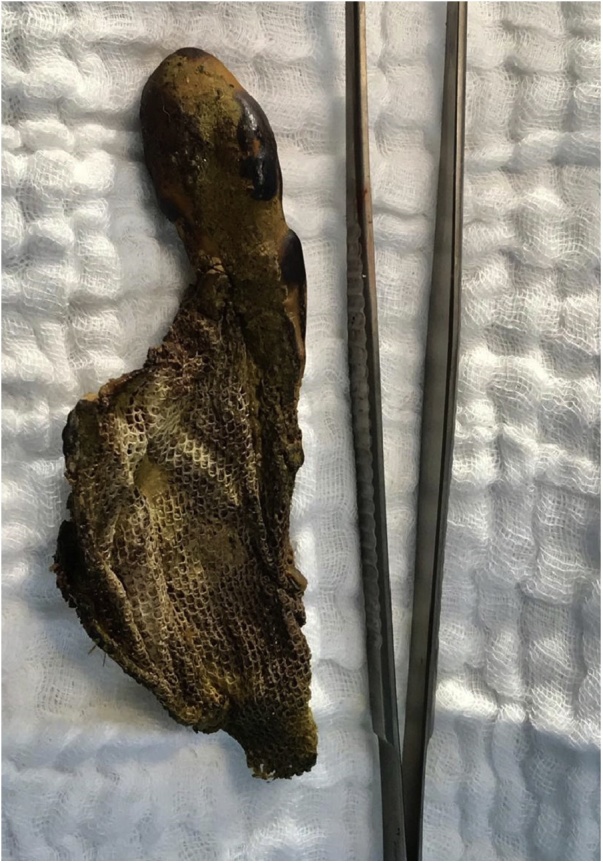
Removal of the mesh plus sigmoidectomy.

A 100-year-old male patient, presented to the emergency department with a 4-day history of abdominal pain, mainly localized in the left quadrants, associated with anorexia and impaired mental status. No nausea nor vomiting were present. The patient only had as previous abdominal surgery in his medical history a TAPP repair of left inguinal hernia 4 years previously with a light weight mesh of polyester. The patient was hemodynamically stable and apyretic. The abdomen was distended, showing generalized tenderness, with physical signs of peritoneal irritation. Laboratory investigation was abnormal with acidosis and elevated lactate. Abdominal CT scan confirmed the presence neumoperitoneum with a strange body into de left colon lumen. These CT features raised suspicion of an area of colon perforation (Fig. 1). Laparoscopy confirmed generalized fecal peritonitis. Exploratory laparotomy was performed. The sigmoid colon was found firmly adhered to the abdominal wall in the left groin region. We identified the presence of left-sided hernia repair mesh penetrating the sigmoid colon from the preperitoneal space (Fig. 2). The mesh material was into the lumen of the sigmoid colon. Obvious sinus or fistula was found between the abdominal cavity, mesh and colon lumen. Sigmoidectomy with removal of the mesh was performed (Fig. 3). Drainage of the generalized fecal peritonitis. Open abdomen plus negative wound pressure therapy. Damage control surgery for the abdominal sepsis. Second look in 48 h with hartmann colostomy and closure of the abdominal wall. 5 days of intensive care unit (ICU) with a ventilator associated pneumonia, acute kidney injury, acute respiratory distress syndrome and multiorganic failure, finally dies on day 7 in the ICU. Pathological analysis of surgical specimen confirmed the substance of the foreign body within the bowel wall along with adjacent inflammatory granulation tissue formation.

## 3 Discussion

Acute abdominal pain (generally defined as pain of less than one week’s duration) is a common presenting complaint among older patients. Approximately one fourth of patients who present to the emergency department are older than 50 years. Older patients tend to present later in the course of their illness and have more nonspecific symptoms. In addition, a broader differential diagnosis must be considered in older patients with abdominal pain that can make an accurate diagnosis more difficult, like in our case with a centenary patient [1,7,9,10].

Incidence rates for mesh erosion, migration and perforation of adjacent organs such colon sigmoid remain unknown. Depending on the different positional relationship of migrating mesh with visceral organs, clinical manifestations vary significantly and may present from 1 to 20 years after inguinal hernia repair. The intestine and urinary bladder were involved in most cases of mesh migration reported from 2003 to 2017. A relatively rare case of a migrated mesh in the retroperitoneal region mimicking a cystic adnexal mass was also documented previously [1,2,[10], [11], [12]].

Lower abdominal pain and mild tenderness were described in the majority of cases, while weight loss, anorexia, symptoms of bowel obstruction, palpable abdominal mass were merely referred to by a few reports. In our case, the male patient complained of severe abdominal pain plus peritoneal irritation signs and acidosis with elevated lactate, simulating a mesenteric ischemia which led to diagnosis delay [3,4,[13], [14], [15]].

Typical signs caused by mesenteric ischemia or peritonitis were almost absent on the computed tomography (CT) but this image showed neumoperitoneum as a sign for gastrointestinal perforation. In previously reported cases, migrating mesh plugs were neglected or misdiagnosed as a poorly defined mass, an intra-abdominal neoplasm or sigmoid diverticulosis based on radiological investigations [[5], [6], [7],[16], [17], [18], [19]].

Incomplete peritoneal repair, inadequate fixation or inappropriate amount of implantation space are possible reasons accounting for mesh migrating into intraabdominal viscera, occasionally followed by fistulas formation or mechanical bowel obstruction. In addition, the sharp edges of prosthetic mesh or tackers could injure the viscera serosal layer, initiating the intraabdominal inflammatory process and subsequent mesh erosion. The bowel injury incidence rate ranged between 0.4 % and 5.6 % in previous studies [[7], [8], [9], [10],[20], [21], [22]].

In our case, the patient presented in the acute phase of a chronic process due to the folling factors: (1) The foreign body reaction to mesh enables gradual movement of the mesh through the anatomic planes in the abdominal cavity; (2) the mesh can be encapsulated by the omentum during its migration and create a channel into hollow organs along with inflammatory reaction and peristaltic bowel movement; (3) Gram-positive cocci are generally responsible for superficial wound infection and can further trigger the deeper infection. Bacterial biofilm can develop over the mesh due to chronic contamination by staphylococcus species, which results in painless mesh migration through the tissue and (4) Prosthetic mesh material decreases the formation of the mesothelial cell layer in peritoneal repaired defects, predisposing the irregular surface of mesh to be surrounded by scar tissue, all 4 processes failed when the mesh cross the entire wall of the colon, introduce into the lumen and generate a free perforation [[17], [18], [19],[23], [24], [25]].

The exact etiology of the complication encountered in our patient remains unclear. During the initial surgery, the mesh should be sutured to the abdominal wall wiht non absorbable tackers, and then the peritoneum can be reconstructed with interrupted tackers to cover the mesh plug. When both of these sutures are insecure, the mesh would migrate into the peritoneal cavity and erode into the sigmoid colon. Al Subaie et al. presumed that the pressure necrosis resulting from the physical contact between the mesh and the colon lead to fistula formation and consequently to mesh migration into the colon. D’Amore et al. found that 5 of 7 patients (2 involving cecum and 5 involving left colonic after plug repair) suffered from diverticular disease and thought that diverticular inflammation might induce the plug to erode into the colon [[11], [12], [13], [14],[23], [24], [25]].

Morbidity and mortality among older patients with abdominal pain are high; evaluation and management often requires admission to the hospital and surgical consultation. In retrospective studies, more than one half of older patients presenting to the emergency department with acute abdominal pain required hospital admission, and 20–33 percent required immediate surgery, like in our case. Surgical intervention occurs twice as often in older patients when compared with a younger population. Overall mortality rates from retrospective series vary from 2 to 13 percent. In a retrospective study of 380 older patients with acute abdominal pain, the presence of free air on plain film radiographs or CT, leukocytosis with a high neutrophilic band count, and age older than 84 years were associated with an increased risk of death, like in our case [[1], [2], [3], [4], [5], [6],^23^,^24^].

Moorman and Price believed that lack of fixation of mesh predisposes to its migration. D’Amore et al. considered that other technical details, such as to avoid the excision of the sac, identify and repair any hole in the peritoneal sac, use preshaped devices, and choose the proper size and light material, could reduce the incidence of this complication. To treat and prevent further erosion of migrating mesh and preserve the function of affected viscera, total removal of the mesh via laparoscopy or laparotomy is advised in clinical practice, along with either partial or entire resection of the organ, like in our case with extraction of the mesh plus enbloc sigmoidectomy. Meanwhile, the possible wound sinus or enteric fistulas linked to the mesh should be completely eradicated by excision in combination with medication therapy (antibiotics, somatostatin and parenteral nutrition). Regardless of the type of mesh repair (open or laparoscopic), meticulous care for correct placement and reliable suture is necessary to avoid complications. Suturing the mesh to the surrounding fascia is a critical step during the operation. Tailoring the mesh, appropriate suture placement and adherence to principles of antisepsis during hernia repair surgery are crucial in avoiding longterm mesh-related complications [[4], [5], [6],[23], [24], [25], [26]].

## 4 Conclusions

TAPP is a safe procedure for treat groin hernias, unless, mesh complications like foreign body reaction, deep-seated infection, mesh migration and perforation into viscera can occur even 20 years later of the procedure with no defined incidence. We recommend do not forget this possible mesh associated issues in a patient with previous TAPP procedure and a foreign body into the lumen of the colon in the abdominal CT.

## Sources of funding

Nothing to declare.

## Ethical approval

The study is exempt from ethnical approval in our institution.

## Consent

Written informed consent was obtained from the patient for publication of this case report.

## Author contribution

Dr. Fajardo, Dr Diaz and Dr Cabrera: Evaluation and post-operative managementof the case along with surgical assistance.

Dr. Fajardo, Dr Diaz and Dr Cabrera: Performed the surgical technique.

Dr. Pedraza, Dr Cabrera: Assisted the surgical procedure.

## Registration of research studies

N/A.

## Guarantor

Mauricio Pedraza Ciro.

## Provenance and peer review

Not commissioned, externally peer-reviewed.

## Declaration of Competing Interest

Nothing to declare.
